# Pediatric spinal ependymomas: an unpredictable and puzzling disease. Long-term follow-up of a single consecutive institutional series of ten patients

**DOI:** 10.1007/s00381-014-2491-7

**Published:** 2014-07-31

**Authors:** Tryggve Lundar, Bernt Johan Due-Tønnessen, David Scheie, Petter Brandal

**Affiliations:** 1 Department of Neurosurgery, Oslo University Hospital, Postboks 4950, Nydalen, 0424 Oslo, Norway; 2 Department of Pathology, Oslo University Hospital, Oslo, Norway; 3 Department of Oncology, Oslo University Hospital, Oslo, Norway

**Keywords:** Pediatric spinal ependymomas, Myxopapillary ependymoma, Anaplastic ependymoma, Gross total resection

## Abstract

**Methods:**

Ten consecutive children (0–18 years) who underwent primary tumor resection for a spinal ependymoma between 1980 and 2011 were included in this retrospective study. Gross motor function and activities of daily life were scored according to the Barthel Index.

**Conclusion:**

Three out of six pediatric patients treated for spinal myxopapillary ependymoma are disease-free after 11 to 33 years of follow-up. The other three have progressive disease, after relapses occurring after 4.5, 7, and 20 years, respectively. One out of two patients with grade II ependymoma had progressive disease from 10 years after initial surgery but is in full-time work in spite of widespread metastatic disease after 32 years. One of the two children with grade III tumor died from progressive disease 17 years from primary diagnosis, while the last one is tumor-free after 19 years. The quality of life is good for three of the four patients with widespread disease, and they are managed conservatively aiming at symptomatic treatment intervention if necessary. We strongly advocate lifelong follow-up for children treated for spinal ependymomas.

Ependymoma is the third most common central nervous system (CNS) tumor in childhood, most often located intracranially, with a predilection for the posterior fossa [15, 18, 28]. The handling of children with such tumors is challenging because of a marked tendency of local recurrence as well as disseminated disease in some patients. Postoperative radiotherapy has therefore often been offered to patients over the age of 3, and in later years, chemotherapy has also been given to pediatric patients with ependymoma [1, 3]. Spinal ependymomas are exceedingly rare in children and adolescents in contrast to adults. In adults, a spinal localization is the most common site of ependymoma and long-term results are very good, even with surgical resection alone [14, 31].

Herein, we summarize our institutional experience of spinal pediatric ependymoma presenting the ten consecutive pediatric cases treated between 1980 and 2011.

## Methods

We retrospectively analyzed a consecutive cohort consisting of ten patients aged 18 years or younger who underwent primary resection for a spinal ependymoma during the years 1980 to 2011 in the Department of Neurosurgery, Oslo University Hospital, Norway.

The cases were collected by reviewing surgical protocols of the relevant time period identifying pediatric ependymoma cases. The histological specimens were reviewed for all 10 cases.

The case record data included sex, age at the time of primary tumor resection, detailed information on repeat operations, and adjuvant treatment.

Scholastic outcome was simplified into normal versus special schooling, and employment attendance into open, sheltered, or no work.

The Barthel Index score is a well-established and validated scale using ten variables to measure performance in basic activities of daily living (ADL) primarily related to personal care and mobility [17]. Scores range from 0 to 100 and a higher score denotes greater independence. The purpose of reporting the Barthel Index was to assess functional status and illustrate possible differences among subgroups within our cohort.

## Results

### Clinical data

Table 1 shows that six children (five boys) had a myxopapillary ependymoma. Two children had ependymoma grade II and two had anaplastic ependymoma, grade III. The sex and age of the child at primary surgery are listed in Table 1.

**Table 1:** Summary of clinical data for the ten patients

Patient	Sex/age	Histology	Localization	Radicality	Radiotherapy	Recurrence	Follow-up	Status
1	M/1.5	Myxopapillary WHO 1	T10–L1	Yes	No	No	27 years	NED
2	M/7	Myxopapillary WHO 1	L1–L3	Yes	No	No	11.5 years	NED
3	M/12	Myxopapillary WHO 1	L2–S3	No	54 Gy			
			T2–5	No	54 Gy	Yes, 7 years	33 years	AWD
4	F/15	Myxopapillary WHO 1	L2	No	52 Gy			
			Local	No		Yes, 20 years		
			Local	No		Yes, 22 years		
			Local	No		Yes, 23 years		
			Paraspinal			Yes, 24 years		
			Paraspinal			Yes, 25 years		
			Pulm. met			Yes, 31 years	32 years	AWD
5	M/16	Myxopapillary WHO 1	L3	Yes	No			
			L3–S5	No	54 Gy	Yes, 4.5 years	6 years	AWD
6	M/18	Myxopapillary WHO 1	L2	Yes	No	No	13 years	NED
7	M/9	Ependymoma WHO 2	Cauda eq.	No	44 Gy	No	33 years	NED
8	F/15	Ependymoma WHO 2	L2	Yes	No			
			Suprasellar		No	Yes, 10 years		
			L2–L3		51 Gy	Yes, 14.5 years		
			Multiple intra-					
			Cranial	No surgery		Yes, 32 years	32 years	AWD
9	M/7	Anaplastic WHO 3	L1–S4	Yes	No			
		Anaplastic WHO 3	Local	No	54 Gy	Yes, 3,5 years		
			Local	No		Yes, 13 years		
			Local	No		Yes, 14 years		
			Local	No		Yes, 15 years		
			Paraspinal	No		Yes, 16 years		
			Paraspinal	No		Yes, 17 years	17 years	DOD
10	F/8	Anaplastic WHO 3	L2–L3	No	54 Gy	No	19 years	NED

*DOD* dead of disease, *AWD* alive with disease, *NED* no evidence of disease

Most patients presented with severe low back pain and varying degrees of paraparesis; in one case, also severe incontinence. The diagnosis of an intraspinal tumor was done by myelography (before 1987) or MRI (from 1987), and from 1987, repeat MRI scans were introduced in the follow-up program. All tumors were intradurally located distal to the vertebra T10 with affection of the conus and/or cauda equina at the primary presentation.

### Pathology

The histological examination revealed myxopapillary ependymoma (WHO grade I) in six patients. Two children were diagnosed with ependymoma WHO grade II and the last two patients had an anaplastic tumor, WHO grade III.

### Primary treatment

All patients had primary surgery. Whenever possible, gross total resection (GTR) was the aim of the surgical procedure, and the degree of resection was evaluated by the surgeon in the first part of the period and in later years also by immediate postoperative MRI scans. The resection was considered complete in six children (Table 1.) All ten patients recovered rapidly after surgery. All four children with incomplete resection were given postoperative local radiotherapy with doses ranging from 44 Gy (one patient) to 51–54 Gy. No patient received systemic antineoplastic treatment.

### Recurrent disease in children with myxopapillary ependymoma

Local tumor recurrence was observed in three of the six patients, after 4.5, 7, and 20 years, respectively, from primary treatment. One of these (Table 1, patient 5) patients with GTR (and no initial radiotherapy) experienced local recurrence after 4.5 years. He underwent repeat surgery and radiotherapy and has later on received extended spinal radiotherapy due to disease in progress. Furthermore, two patients with non-GTR who did receive primary radiotherapy have experienced recurrent disease (Table 1).

In one patient with recurrence after 7 years (Table 1, patient 3), widespread dissemination to the cervicothoracic spinal canal was diagnosed synchronously to local progression. The clinical presentation at the time of recurrence was a high thoracic transverse myelopathy, and the patient had a tumor resection at the level of T2–5, followed by adjuvant radiotherapy above the primary lumbosacral level which had been irradiated in the primary situation. He was in full-time work as a blacksmith until a couple of years ago but is now, 26 years following his recurrence, outside the labor market because of severe back pain. A recent MRI has demonstrated multiple tumor manifestations from C6 and distally in the spinal canal. Clinically, however, in spite of his severe back pain, he is neurologically remarkably well, with preserved sexual, bladder, and bowel function.

The only girl in this group (patient 4) underwent tumor resection at the L2 level and classified as non-GTR had received 52 Gy locally. Twenty years later, she came back with local recurrence. During the subsequent 11 years she has undergone five resections for intraspinal or paraspinal tumor progression and 31 years after initial treatment also resection of two pulmonary metastases from her myxopapillary ependymoma. She has also had oncological treatment with chemotherapy and further radiotherapy.

### Recurrent disease in children with ependymoma grade II and III

One of the two patients with grade II tumor is disease-free with 33 years follow-up. The other had recurrence and it is a bit surprising that the one with recurrence was a GTR primarily, whereas in the non-GTR patient, the disease did not recur. The latter had radiotherapy up front. The same peculiar situation was found for grade III tumors; the one with GTR had recurrence, whereas the one with non-GTR and radiotherapy did not experience regrowth of the tumor.

The grade II patient with recurrence came with clinical symptoms of a suprasellar tumor 10 years after primary resection (GTR) of a grade II ependymoma at the L2 level at the age of 15 years (Table 1, patient 8). After resection (GTR) of the intracranial grade II ependymoma, further follow-up disclosed local recurrence at the level of L2/3 5 years later. She received radiotherapy (51 Gy) at the lumbar level. Today, 32 years after initial treatment, she has multiple intraventricular manifestations of her disease, but with sparse clinical symptoms.

One of the children treated for grade III tumor is disease-free 19 years after a non-GTR resection followed by local radiotherapy (54 Gy). The other grade III patient did not receive initial radiotherapy after a GTR resection. He succumbed to his neoplastic disease 17 years after initial surgery; he had a total of seven tumor resections (Table 1, patient 9).

### Motor function and activities of daily life

Gross motor function and management of the activities of daily life is good in all the nine survivors. In terms of the Barthel Index, the score is 100 in eight and 80 in the last one.

### School, education, and work

One of the nine survivors has a current age below 20 (18 years) and follows a regular school program. The remaining eight survivors are aged 22–47 years. One is a student and six are in regular work, whereas the last two are outside the labor market.

## Discussion

Spinal ependymomas are exceedingly rare in the pediatric population when compared to adults, where a spinal location is the most frequent localization of ependymoma [4, 9, 20, 31]. In a recent publication from our institution, 86 consecutive spinal ependymoma cases diagnosed between 1987 and 2007 were reviewed. The results were good with a 10-year overall survival (OS) of 91 % and a progression-free survival (PFS) of 75 %. Only two of these cases were children [14].

In a review article, Oh and coworkers found that the prognosis for adults treated for spinal cord ependymoma varied with tumor location within the spinal canal; a better prognosis was found for patients with tumors in the upper spinal cord than for patients with a distal tumor location [23]. All WHO grades were included, also myxopapillary tumors (grade I).

In another review performed by the same authors, a similar trend was found in children; a better prognosis for individuals with tumors in the upper spinal canal compared to a distal tumor location [24]. In this report, however, myxopapillary ependymomas were excluded. Furthermore, the vast majority of pediatric spinal ependymomas occur in the distal spinal canal [32].

A number of authors have pointed out that the long-term prognosis for pediatric spinal ependymoma cases may be inferior compared to adult cases [6, 11, 13, 19, 32].

Because of the limited numbers of such cases in the English literature, as well as variability of histological grades, different treatment strategies have been undertaken and the optimal treatment schedules have not been firmly established [6].

Although total surgical resection has been shown to improve long-term results [30] and reduce the need for postoperative radiotherapy at least in grade I and II tumors, some authors have advocated local postoperative irradiation following incomplete resection in all children with anaplastic (grade III) lesions irrespective of the completeness of resection [3, 13].

McGuire and coworkers do not, based on compiled data from 55 children, recommend postoperative radiotherapy for pediatric spinal ependymomas. This is in contrast to their recommendations for pediatric posterior fossa ependymomas where they show an improved 5-year survival with postoperative irradiation after surgery based on analysis of 193 cases treated between 1973 and 2003 [19].

Gross total resection has been shown to reduce the risk for recurrence and increase progression-free survival (PFS) [5, 7, 30]. In cases with involvement of the conus, the likelihood of complete resection is reduced or implies an increased risk of postoperative morbidity [2].

Several authors state that the myxopapillary tumor of the spinal canal appears to have a marked tendency to recur and metastasize [6, 11, 13, 27]. Therefore, many authors advocate postoperative radiotherapy [8, 25, 26].

Pica et al. analyzed 85 cases of spinal myxopapillary ependymomas of all ages treated between 1970 and 2007 and found significantly improved 5-year PFS with concomitant local radiotherapy. Their series appears to include mainly adult cases, with a median age of 37 years. Median follow-up was only 60 months [29].

Al-Halabi et al. advocate routine postoperative local irradiation for pediatric spinal myxopapillary ependymomas, based on a study of seven cases treated between 1985 and 2008 [3]. Again, the follow-up period is relatively short, with a median of 78 months.

Based on this, it is evident that long-term follow-up over the decades has not previously been reported and most published series are small. Compiled data from several centers have been published for 85 children operated between 1970 and 2007 for spinal myxopapillary ependymoma; however, with short follow-up times (see above) [29].

Even in adults, very long-term results are absent. Gomez and coworkers report high failure rates in 37 patients treated between 1955 and 2001, but the median long-term follow-up was 121 months [13].

The present series include all children treated for a spinal ependymoma in our institution in a time period of 31 years. They represent 0.6 % of all children and adolescents treated for a CNS tumor in the same time period and 20 % of the pediatric ependymomas. There was no surgical mortality in this series, and the observed long-term survival is very encouraging.

Widespread dissemination within the spinal canal and even intracranially from spinal ependymoma has been reported several times [11, 21]. Although intracranial spread from a spinal ependymoma has been published previously, it seems to be very unusual. Such single Norwegian pediatric case was published in 1980; a 12-year-old boy who deceased from intracranial tumor dissemination 6.5 years after treatment of the primary spinal ependymoma [21].

Paraspinal progressive disease and even metastatic disease is also well known, although rare [10, 12, 16, 22].

Fassett et al. reported intracranial manifestation in four out of five pediatric cases with spinal ependymoma treated inside an 11-year-period [11]. They therefore advocate craniospinal MRI of all pediatric ependymoma cases. We agree with Stephen and coworkers, who advocate prolonged follow-up regimens including MRI for pediatric cases with spinal ependymoma, especially patients with myxopapillary tumors [32]. Our series demonstrate recurrent disease also in patients with grade II or III tumors.

We consider our small series of ten patients representative, since it is consecutive and includes all children operated for a spinal ependymoma in a period of 31 years. The recurrence rate is high (50 %) and it should be noted that two of the recurrences were diagnosed as late as 14 and 20 years following the time of primary diagnosis.

It was the reappearance of severe clinical symptoms (pain and/or paraparesis) which led to the diagnosis of recurrence. MRI was used for diagnosis and repeat surgery was performed (in all but one patient) after local recurrence had been demonstrated. All five have undergone local radiotherapy. Four of them now have more widespread tumor manifestations in the spinal canal but for the time being have a relatively stable or very slowly progressive situation with respect to their neoplastic disease in the spinal canal. For one of them, this is the situation 26 years after the second surgery and further radiotherapy for progressive disease in the upper thoracic level, 7 years after initial surgery and postoperative radiotherapy in the lumbosacral region.

One patient has been operated for intracranial ependymoma as well as for a local recurrence. In this patient, the widespread intracranial manifestations appear indolent after further 22 years of follow-up and she is in full-time work 32 years after the primary surgery.

Our recurrence rate of 50 % is high and above the rate in many other reported small series and even in compiled data from many series. Because of the relatively short follow-up in most series, as well as the late recurrences we have observed in the present study, it is possible that lower rates are merely a consequence of too short follow-up. Furthermore, the intriguing data presented by Fassett and coworkers, with simultaneous diagnosis of intracranial ependymoma lesions in four out of five children at the time of primary diagnosis of a spinal myxopapillary ependymoma, open another question [11]. Is it obvious that further manifestations found in the spinal canal or intracranially in the long-term follow-up are metastatic disease from the primary ependymoma in the lumbosacral region?

Since we do not have complete MRI data, especially for the patients operated 30 years ago, we can only conclude that consequent implementation of craniospinal MRI over time would be very valuable in such patients.

Because of the puzzling indolent clinical situation observed in at least two of the patients with widespread disease and more than 30 years follow-up seen in this series, it is evident that it is difficult to make treatment decisions for this patient group. Furthermore, very long-term follow-up with MRI seems indicated in these patients.

## Conclusion

Three out of six children treated for distal spinal myxopapillary ependymoma have not experienced recurrent disease and are free of tumor as evaluated by MRI after 11 to 33 years of follow-up. Three children had surgery for local recurrence and/or distant spinal disease from 4.5 to 20 years after the initial surgery. The patient with very late recurrence has also had multiple surgeries for paraspinal progression, as well as for metastatic disease to the lung, but is still alive after 32 years.

In one of the two patients treated for grade II tumor, widespread intracranial and distant spinal disease have been evident for 18 years, but the patient is in full-time work at the age of 48; 32 years after the initial treatment.

One of two patients with grade III tumor died after 17 years due to progressive paraspinal manifestations. The last one is tumor-free after 19 years follow-up. GTR may cure children with distal spinal ependymoma, but does not prevent recurrence in all cases. Incomplete resection followed by local radiotherapy can also give excellent long-term results, but recurrence may appear after many years. Long-term follow-up including MRI for decades is therefore necessary.
